# Visual evoked potentials in offspring born to mothers with overweight, obesity and gestational diabetes

**DOI:** 10.1371/journal.pone.0203754

**Published:** 2018-09-12

**Authors:** Francisco J. Torres-Espínola, Staffan K. Berglund, Salomé García, Miguel Pérez-García, Andrés Catena, Ricardo Rueda, Jose Antonio Sáez, Cristina Campoy, for the PREOBE team

**Affiliations:** 1 Centre of Excellence for Paediatric Research EURISTIKOS, University of Granada, Granada, Spain; 2 Department of Paediatrics, University of Granada, Granada, Spain; 3 Department of Clinical Sciences, Pediatrics, Umeå University, Umeå, Sweden; 4 Clinical Service of Neurophysiology, Clinical University Hospital San Cecilio, Granada, Spain; 5 Mind, Brain and Behaviour International Research Centre (CIMCYC), University of Granada, Granada, Spain; 6 Department of Personality, Neuropsychology and Behavior, University of Granada, Granada, Spain; 7 Department of Experimental Psychology, University of Granada, Granada, Spain; 8 Scientific Department of Abbott Nutrition, Granada, Spain; 9 CIBERESP: Spanish National Network in Epidemiology and Public Health, Institute Carlos III Granada’s node, Granada, Spain; Centre Hospitalier Universitaire Vaudois, FRANCE

## Abstract

**Background:**

Overweight, obesity, and gestational diabetes (GD) during pregnancy may negatively affect neurodevelopment in the offspring. However, the mechanisms are unclear and objective measures of neurodevelopment in infancy are scarce. We hypothesized that these maternal metabolic pathologies impair cortical visual evoked potentials (cVEPs), a proxy for visual and neuronal maturity.

**Design:**

The PREOBE study included 331 pregnant women stratified into four groups; normal weight (controls), overweight, obesity, and GD (the latter including mothers with normal weight, overweight and obesity). In a subsample of the offspring at 3 months (n = 157) and at 18 months (n = 136), we assessed the latencies and amplitudes of the P100 wave from cVEPs and calculated visual acuity.

**Results:**

At 3 months of age, visual acuity was significantly poorer in offspring born to GD mothers. At 18 months of age, there were no differences in visual acuity but infants born to GD mothers had significantly longer latencies of cVEPs when measured at 15’, and 30’ of arc. The group differences at 30’ remained significant after confounder adjustment (mean [SD] 121.0 [16.0] vs. 112.6 [7.6] ms in controls, p = 0.007) and the most prolonged latencies were observed in offspring to GD mothers with concurrent overweight (128.9 [26.9] ms, p = 0.002) and obesity (118.5 [5.1] ms, p = 0.020).

**Conclusions:**

Infants born to mothers with GD, particularly those with concurrent overweight or obesity, have prolonged latencies of visual evoked potentials at 18 months of age, suggesting that this maternal metabolic profile have a long lasting, non-optimal, effect on infants´ brain development.

## Introduction

The rates of overweigh and obesity have experienced exceptional growth and become an increasing public health problem. Following this epidemic, numerous studies are currently exploring how these metabolic pathologies affect human health.[1] One important research field is the studies exploring the effect of overweight and obesity on pregnant women and their offspring. It is known, that increased maternal weight before pregnancy, and rapid weight gain during pregnancy, both constitute risk factors for development of gestational diabetes (GD) and other gestational complications in the mother. [2] Furthermore, these conditions have also been associated with impaired growth and neurodevelopment of the offspring, even at long term. Early programmed adverse effects on body composition, metabolic, and mental performance have been suggested.[3–12] However, these associations have been difficult to confirm or reproduce, since precise and objective methodologies for neurodevelopment assessment during infancy are scarce.

Measurement of cortical visual evoked potentials (cVEPs) is a neurophysiological technique that can provide objective information about the function of the visual system in infants and children too young to communicate visual symptoms or cooperate in the standard assessments of visual function.[13] cVEPs have been suggested as a promising measure for the neurological evaluation of visual function, and also a proxy for general neurodevelopment. The latencies of the cVEP are closely correlated to the process of neuronal myelination that occurs during the first 1–2 years of postnatal life.[14–16] Some studies have reported that infants born to mothers with diabetes mellitus type I and type II have impaired latencies and amplitudes of cVEPs.[17, 18] However, we found no previous studies exploring, the separated effect of overweight, obesity and GD in patient without pre-gestational diabetes.

The objective of this study was to explore the cVEPs in offspring born to mothers with overweight, obesity and GD, and compare to children born to healthy normal weight controls. We hypothesized that these maternal metabolic alterations would negatively affect the cVEPs in the offspring at 3 and 18 month of age.

## Methods

### Study design and participants

The PREOBE study is a prospective mother-child cohort study, conducted between 2007 and 2012 (registered in www.ClinicalTrials.gov) with the purpose of studying the effects on pregnancies and offspring of PRE-gestational OBEsity, overweight and GD. The design of the study has been published elsewhere.[19] In brief, 331 pregnant women with singleton pregnancies and age between 18 and 45 years were included between 12 to 20 weeks of pregnancy (occasionally until 34 weeks). The mothers were stratified into four different groups based on their calculated pre-gestational body max index (BMI) and GD condition: Healthy normal weight group (18.5 kg/m^2^ ≤BMI<25 kg/m^2^; n = 132), overweight group (25 kg/m^2^ ≤BMI<30 kg/m^2^; n = 56), obese group (BMI≥30 kg/m^2^; n = 64), and GD group (BMI≥18.5 kg/m^2^; n = 79). The group allocation was performed at 34 weeks of gestation where all mothers with GD diagnosed at any stage of pregnancy were allocated to the GD-group, independently of BMI. Consequently, after such re-distribution, the GD included 23 with overweight, 24 with obesity, and 32 with normal weight.

**Fig 1:**
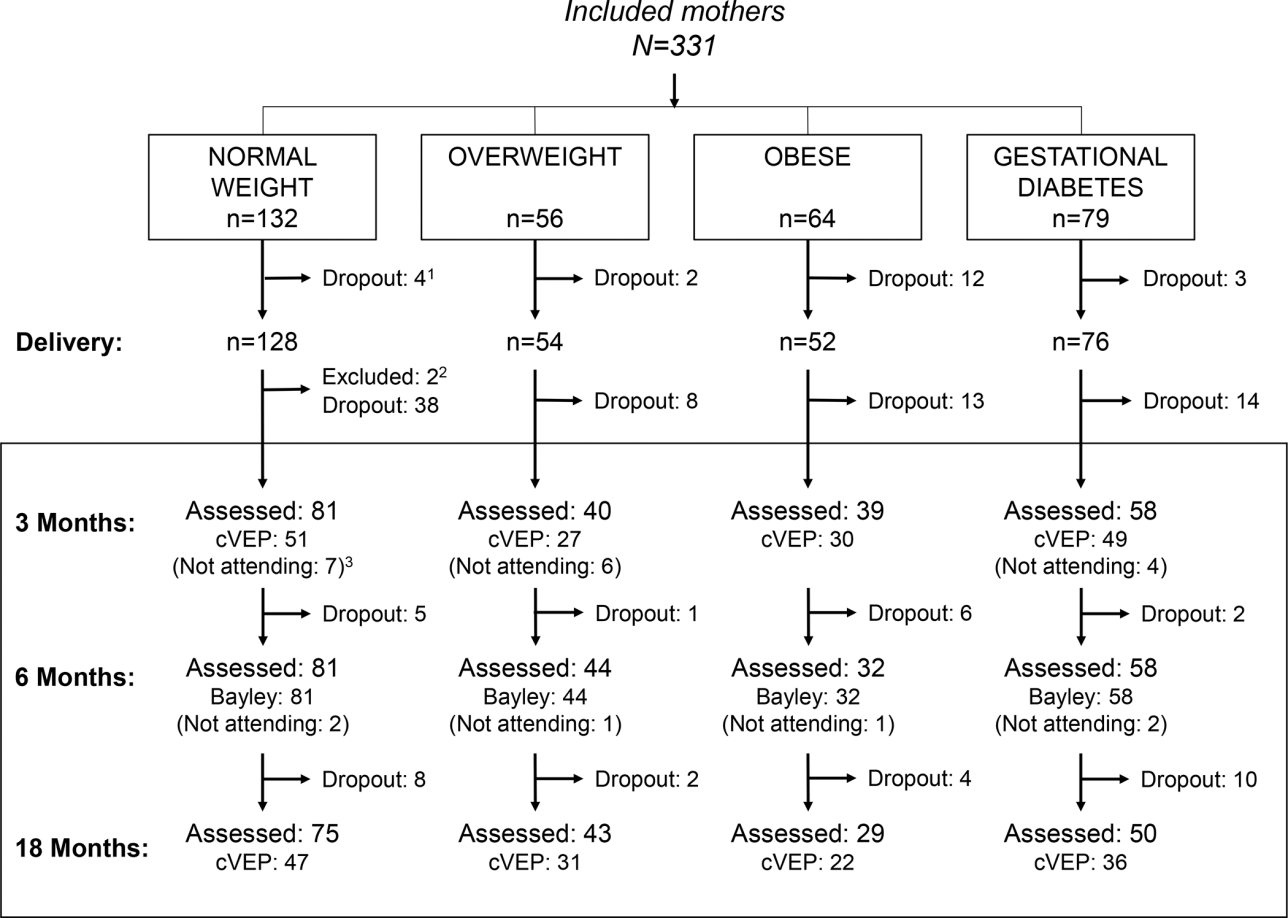
Study profile. ^1^ Of the 331 included mothers, 21 dropped out of the study before delivery and another 73 before the first neurodevelopmental follow up at 3 months of age. ^2^ Two mother-child pairs were excluded after delivery due to congenital disorders. ^3^ Seventeen mother–child pairs at 3 months and six at 6 months did not show up at the assessments but remained in the study for later visits, those are described as “not attending”.

The exclusion criteria were: simultaneous participation in any other research study or any of the following diseases; pre-gestational diabetes, hypertension or preeclampsia, fetal intrauterine growth retardation, maternal infection during pregnancy, hypo/hyperthyroidism, hepatic diseases and renal disease), and vegan diet. In the present analyses, another 2 cases were excluded after delivery due to congenital disorder in the offspring (Fig 1).

### Ethical statement

The research was approved by the Bioethical Committees for Clinical Research of the Clinical University Hospital San Cecilio and the Mother-Infant University Hospital of Granada. An ethical approval was also obtained by the Research Bioethical Committee of the University of Granada. Written informed consent was obtained from all mothers and/or tutors at their offspring follow-up study entry.

### Data collection

As a part of the original study design, information regarding maternal age, pre-gestational weight, maternal educational level, parity, smoking habits during pregnancy, marital status and maternal intelligence quotient (IQ) were obtained at inclusion and all mothers were assessed at 24, at 34 weeks, and at delivery, including measures of iron status and glucose. We also registered information regarding the newborn child, including gestational age, sex, anthropometrics and cord blood laboratory status.[19]

In the present neurodevelopmental follow up study, the mother–infant pairs were called back for follow-up visits at 3, 6 and 18 months of age including cVEPs (3 and 18 months), neuropsychological testing (6 and 18 months), anthropometric measures and health questionnaires. The three preterm babies were assessed at corrected age.

### Cortical visual evoked potentials

At 3 months of age we were able to evaluate cVEPs in 157 infants (Fig 1). Apart from the two excluded cases (congenital disorder), 73 participants dropped out after delivery, 17 infants remained in the study but their parents decided not to participate the follow up at 3 months, and 61 cases came to the evaluation but the cVEP measure failed because the child could not be calmed. In one of the cases at 3 months, we only registered latencies and in another one only the amplitudes, resulting in 156 cases analyzed for each outcome. Moreover, at 18 months of age, another 38 had dropped out and successful measures of cVEP were performed in 136 of the 197 infants assessed (S1 Table). The reasons for drop out during the follow up period between delivery and 18 months was not monitored in detail and most drop outs did not declare their reasons.

Infants’ cVEPs were recorded in a partially darkened room (mean background light 0.15 ft-Lamberts; dark adaptation for 20 minutes) in awake condition (without sedation). Two caps of two different sizes (38–42 cm at six months and 42–46 cm at eighteen months) with electrodes placed according to the 10–20 system were used (*Electro-Cap International including*: Fz as reference, O1, Oz and O2 as actives [Oz on inion, O1 3cm on the left and O2 3 cm on the right] and Cz as ground electrode). [20] cVEPs were obtained in a quiet room under controlled conditions while the participants were aware, alert and placed at the same height as the stimulation screen. If the baby did not keep attention, then the test stopped and only began when attention came back. The cVEPs in infants were registered using a Schwarzer topas EMG System, (NATUS, California, USA). The visual stimulus was a reversal pattern of black and white checkerboard (contrast 100%) generated on a CRT monitor. Stimulus were performed in a shape of binocular frequencies at 2°, 1°, 30 ', 15' and 7½'. The average luminance was 39 kcd/m^2^ and the investment rate was 2.1. Responses were amplified with filter from 1.5 Hz to 100 Hz. As outcome in the present paper we used the P100 wave latencies and amplitudes as suggested by McCulloch and Skarf.[13] Visual acuity was calculated using linear regression between amplitudes and visual angle (transformed to cycles per minutes).[21] Only cases with a regression coefficient above 0.5 were included in the analyses.

### Neurodevelopmental testing

At 6 and 18 months of age, infants’ neurodevelopment were assessed by using the Bayley Scales of Infant Development, Third Edition (BSID-III). All infants were examined by the same trained psychologist (FJTE). The infant evaluation by BSID-III is performed across five domains: *cognitive skills*, *receptive language*, *expressive language*, *fine motor*, *and gross motor development* and a parental questionnaire to evaluate *socioemotional development*. [22]

### Statistical method

All statistical analyses were performed using the SPSS statistical software package for Windows (version 22.0; IBM SPSS Inc., Chicago, IL, USA). Continuous and normally distributed variables were displayed as mean and standard deviation (SD). Differences between the four groups in cVEP were explored using unadjusted analysis of variance (ANOVA) as well as confounder adjusted analyses using multivariate analyses of covariance (ANCOVA). The confounder introduced in the models were gestational age at birth and sex, due to a significant correlation to at least one cVEP outcome and maternal age and maternal education due to significant group differences. The significance level was set to p<0.05. This study was originally powered based on outcomes during pregnancy.[19]

## Results

Table 1 shows the background and baseline characteristics of the mothers and their offspring in all 157 infants evaluated at 3 months, including comparison of these characteristics between groups. We observed significant differences between the study groups in maternal age and there was a non-significant trend of higher educational levels in the control group and in the overweight group compared to the other two. Three cases were born preterm, one born to an obese, one to an overweight, and one born to diabetic mother. No severe complications such as asphyxia were recorded in the analyzed infants.

**Table 1:** Baseline and background characteristics of the mother-child pairs who participated in the cVEPs follow up at 3 months of age (n = 157), including group comparisons among the four PREOBE-groups.

		Normal weight	Overweight	Obese	Gestational Diabetes	p
		n = 51	n = 27	n = 30	n = 49	
Maternal Glucose at 24 weeks (mg/dl)		80.64±19.16	91.54±16.03	88.08±17.13	101.31±27.83*	**0.004**
Maternal Glucose at 34 weeks (mg/dl)		86.21±20.41	89.65±21.28	91.93±17.27	95.01±23.93	0.278
Maternal Glucose at delivery (mg/dl)		80.09±20.04	91.50±24.37	95.85±34.06	98.24±34.23*	**0.028**
Maternal Ferritin at 24 weeks (ng/ml)		23.05±17.25	19.71±12.25	33.73±27.10	25.39±17.83	0.061
Maternal Ferritin at 34 weeks (ng/ml)		18.04±15.37	13.50±7.13	16.04±8.66	21.24±16.47	0.109
Maternal Ferritin at delivery (ng/ml)		27.56±16.12	26.28±17.55	17.05±6.92*	31.23±16.08	**0.014**
Maternal Age (y)		31±7	33±4	30.50±8	34±6*	<0.001
Maternal educational level	Primary/Secondary	44.9%	55.6%	73.3%	65.3%	0.058
	University/Doctor	55.1%	44.4%	26.7%	34.7%	
Marital Status	Single/Separated	2%	0%	6.7%	0%	0.291
	Married/Cohabitating	95.9%	100%	90%	100%	
	Others	2%	0%	3.3%	0%	
Maternal IQ (points)		111±15	104±21	106±22	104±20	0.177
No of siblings	0	59.2%	59.3%	40.3%	55.1%	0.534
	≥1	40.8%	40.3%	56.7%	44.9%	
Smoking	no	83.7%	87%	96%	93%	0.335
	yes	16.3%	13%	4%	7%	
Birth weight (g)		3277±398	3353±482	3468±541	3278±407	0.253
Birth HC (cm)		34.61±1.39	34.6±1.21	34.50±1.64	34.63±1.35	0.987
Gestational Age at birth (wk)		40±1	39±3	40±6	39±5	0.569
Cord Blood Glucose (mg/dl)		68.77±20.90	64.00±19.42	70.16±26.40	73.61±20.85	0.468
Cord Blood Ferritin (ng/ml)		182.41±103.99	177.30±97.45	187.72±90.28	181.26±112.46	0.994
Sex	Boy	46.9%	40.7%	60.7%	55.1%	0.416
	Girl	53.1%	59.3%	39.3%	44.9%	
Infant type of feeding	Breast-fed	57.1%	53.8%	37.9%	42.6%	0.426
	Infant formula	18.4%	19.2%	13.8%	19.1%	
	Mixed	24.5%	26.9%	48.3%	38.3%	

Data are mean ± Standard Deviation and p-values for unadjusted overall group effect using ANOVA for means and Chi-square test for proportions.

*Values significantly different from the normal weight group in a Bonferroni adjusted post hoc test. HC: head circumference.

The results of the cVEPs performed at 3 and 18 months, including a comparison between the four PREOBE-groups are presented in Table 2. At 18 months of age, there were significant group differences in the latencies of P100 at 1° (p = 0.033) and at 30’ of arc (p = 0.003). A similar trend was observed at 15’ (p = 0.053) and 7½’ of arc (p = 0.059). The post hoc analyses demonstrated significantly prolonged latencies in children born to GD mothers compared to those of normal weight mothers in the waves P100 at 30’ of arc (Bonferroni adjusted p-value for infants born to GD vs. normal weight = 0.002) and P100 at 15’ of arc (Bonferroni adjusted p-value = 0.042). In confounder adjusted analyses (P^b^-value in Table 2), the overall group differences remained significant with regard to the latencies obtained at 30’ of arc (p-value for ANOVA = 0.007) and the post hoc test for difference between GD-group and controls. Furthermore, a similar significant group difference in the adjusted model was found regarding the latencies of P100 at 7½’ of arc (p-value for ANCOVA = 0.044).

**Table 2:** Amplitudes and latencies of infant’s P100 visual evoked potentials (cVEPs) at 3 and 18 months of age in children born to mothers with pre-pregnancy overweight, obesity or gestational diabetes compared to those born to healthy normal weight pregnant women (controls).

	Normal Weight	Overweight	Obesity	Gestational Diabetes	p^a^	p^b^
*Latencies at 3 mo (ms)*	n = 51	n = 27	n = 30	n = 49		
P100–2^○^ of arc	115.01±13.94	112.57±8.52	117.66±12.85	117.81±13.71	0.316	0.648
P100–1^○^ of arc	119.55±15.10	117.86±10.32	121.08±13.04	123.09±14.80	0.403	0.799
P100–30’ of arc	125.99±15.18	124.39±15.00	129.13±17.27	130.70±16.69	0.305	0.660
P100–15’ of arc	136.72±19.05	136.36±15.27	140.40±17.89	143.12±15.76	0.272	0.685
P100–7 ½’ of arc	147.70±21.18	145.91±13.43	147.75±16.26	154.67±15.63	0.481	0.811
*Amplitudes at 3 mo (Hz)*						
P100–2^○^ of arc	21.19±12.01	22.77±11.69	23.64±17.79	26.92±13.90	0.246	0.224
P100–1^○^ of arc	21.95±11.16	21.66±10.02	21.58±15.29	24.94±13.88	0.554	0.511
P100–30’ of arc	18.14±9.34	18.15±8.74	16.53±11.32	21.09±11.56	0.254	0.326
P100–15’ of arc	15.16±8.98	15.56±7.06	14.50±8.65	15.63±8.09	0.958	0.834
P100–7 ½’ of arc	8.30±6.22	9.37±6.18	13.60±10.18	9.86±6.39	0.182	0.116
*Latencies at 18 mo (ms)*	n = 47	n = 31	n = 22	n = 36		
P100–2^○^ of arc	106.24±5.76	105.80±7.65	108.08±13.96	109.77±11.26	0.316	0.340
P100–1^○^ of arc	108.66±6.79	109.00±7.20	108.31±6.09	113.10±9.54	**0.033**	0.079
P100–30’ of arc	112.57±7.64	114.71±7.79	113.69±6.00	120.98±16.03*	**0.003**	**0.007**
P100–15’ of arc	119.17±9.11	120.51±13.27	121.67±9.11	126.28±12.82*	0.053	0.088
P100–7 ½’ of arc	127.09±9.52	132.68±10.98	126.91±11.70	132.37±5.27	0.059	**0.044**
*Amplitudes at 18 mo (Hz)*						
P100–2^○^ of arc	22.49±12.43	20.11±10.61	19.63±10.37	21.22±13.08	0.776	0.949
P100–1^○^ of arc	24.56±12.62	21.78±12.53	22.70±13.43	23.49±15.31	0.838	0.892
P100–30’ of arc	21.77±10.60	19.90±12.80	18.24±11.16	20.17±12.58	0.704	0.850
P100–15’ of arc	19.83±10.28	18.93±10.66	15.66±11.93	19.75±12.21	0.534	0.592
P100–7 ½’ of arc	19.09±9.36	15.88±9.70	16.90±11.92	16.35±6.85	0.535	0.696

Data are mean ± Standard Deviation, p^a^-values for unadjusted overall group effect using ANOVA, and p^b^-values for overall group difference adjusted for gestational age at birth, maternal age, infant sex and maternal education using ANCOVA.

*Values significantly different from the normal weight group in a Bonferroni adjusted post hoc test.

To further explore the differences observed in latencies of P100 at 30’ of arc at 18 months of age between infants from the GD group and those from normal weight group, we stratified the diabetic group based on the maternal pre-gestational BMI. Each subgroup of infants born to GD mothers (normal weight, overweight and obese) was compared to the control group with mean (SD) of 112.6 (7.6) ms. We found, in confounder adjusted analyses, the most prolonged latencies in those babies born to overweight (128.9 (26.9) ms, p = 0.002 vs. controls) and obese (118.5 (5.1) ms, p = 0.020) diabetic mothers, while the normal weight diabetic group did not differ significantly (116.6 (6.1) ms, p = 0.140).

Visual acuity could only be assessed in a subsample of the study (Table 3). For those, there was a significant group difference in visual acuity at 3 months of age (p = 0.014). The post hoc test showed that the vision was significantly lower in infants born to GD mothers compared to controls with a logMAR mean difference of 0.19 (95% CI: 0.07–0.31). At 18 months, there were no differences in visual acuity.

**Table 3:** Estimated visual acuity at 3 and 18 months of age in children born to mothers with pre-pregnancy overweight, obesity or gestational diabetes compared to those born to healthy normal weight pregnant women (controls).

	Normal weight	Overweight	Obesity	Gestational Diabetes	p
	n = 33	n = 12	n = 13	n = 29	
Visual Acuity at 3 mo (logMAR)	1.03±0.28	1.09±0.17	1.16±0.19	1.22±0.20*	**0.014**
	n = 21	n = 15	n = 10	n = 15	
Visual Acuity at 18 mo (logMAR)	0.94±0.25	0.96±0.23	0.99±0.19	1.04±0.24	0.618

Data are mean ± Standard Deviation and p-values for overall group effect using ANOVA.

*Values significantly different from the normal weight group in a Bonferroni adjusted post hoc test.

In secondary analyses we used linear regression to assess the relationship between dichotomized cVEP measures (using a median [P50] or third quartile split [P75]), and the 3 main scores of the Bayley III test at 18 months (*language*, *motor function and cognitive function*). All analyses were adjusted for gestational age and infant sex. The regression models revealed significant correlations to composite cognitive scores at 18 months: latencies of wave P100 at 30’ of arc above P75 measured in infants at 3 months of age, correlated significantly to lower cognitive composite score at 18 months (adjusted, unstandardized regression coefficient R [95% CI]: -4.5 [-9.00; -0.069], p = 0.047); and, at 18 months of life, amplitudes of wave P100 at 30’ of arc above P50 correlated significantly to higher cognitive scores (adjusted, unstandardized regression coefficient R [95% CI]: 3.915 [0.209; 7.620], p = 0.039). No correlations were observed between cVEPs and motor or language scores.

## Discussion

In this study, we explored the influence of being born to a mother with overweight, obesity or GD during pregnancy on the brain development using cVEPs as a proxy. While there were no significant differences in latencies and amplitudes obtained in the offspring of non-diabetic overweight or obese women compared to controls, children born to mothers with GD had significantly poorer visual acuity at 3 months and prolonged latencies of cVEPs at 18 months of age. The difference was most pronounced in the subgroups of gestational diabetic mothers who were also overweight or obese, suggesting a negative interaction of these two risk factors. In a secondary analysis we observed that short latencies at 3 months and high amplitudes at 18 months significantly correlated to higher Bayley III scores of cognition, supporting the clinical relevance of cVEPs in assessing infant development.

Maternal diabetes and obesity are common example of early risk factors that may contribute to *“early programming”* of later health and disease as suggested by Barker. [23] These conditions have been associated with poor neurodevelopment in several previous studies, even though the mechanisms are unclear and causality is not yet shown.[17, 24–27] BeBoer et al. [28] showed that offspring born to pregnant women with type I diabetes showed lower Bayley II scores of motor- and cognitive development at 12 months of age. Ornoy et al. [29, 30] found that children born to GD mothers had lower cognitive, gross motor and fine motor development scores at 9 years of age; even more, they reported that they were more likely to develop disorders of attention such as hyperactivity and impulsivity (ADHD). In the Avon Longitudinal Study of Parents and Children (ALSPAC), Fraser et al. [31] concluded that GD is consistently associated with lower cognitive development (a difference up to 5 points in IQ) and low educational levels among the offspring. They also concluded that the exact mechanism behind the association between diabetes and poor neurodevelopment is unclear. The suboptimal metabolic control during GD has been suggested to cause dysfunctions at the cortical level in the brain; this hypothesis is partly supported by previous studies carried out in humans and animals.[32–36] Our results suggest a mechanism that includes impaired neuronal function, since cVEPs are considered a proxy for neuron myelination (latencies) and visual acuity (amplitudes),[37, 38] and are in agreement with studies reported by Brinciotti et al.[18, 39]

If the observation found in this study represents a true causal relationship, it suggests that the hyperglycemic status of GD mothers, have contributed to the observed effects in the offspring, either directly during fetal life or by affecting their postnatal precondition. Since this is an observational study, we can only speculate regarding such mechanisms: During the prenatal phase, the hyperglycemic status of the GD mothers is transferred to the fetus. This was also found in the present cohort where cord blood glucose levels were higher in the offspring to GD mothers compared to the other groups.[19] It has been shown that the fetal pancreas already at 20 weeks of gestational age is capable to respond to this hyperglycemia by increasing insulin secretion and increase the fetal metabolism with up to 30%. Again, this was also likely in the present cohort where cord blood insulin levels were higher in the GM group, even though the differences did not reach statistical significance.[19] It is likely that this state of hyperglycemia, hyperinsulinemia and enhanced metabolism, may have lay ground for a poorer myelination process of the auditory system. For instance, an increased metabolism has been associated with increased risk of fetal hypoxia that follow due to limited oxygen transport through the placenta.[4, 40] With regard to postnatal mechanisms, GD increases the risk of hypoglycemia in the newborn offspring, a condition that has been associated with impaired neurodevelopment in previous studies and may also explain an impaired visual development.[41] Unfortunately, we did not monitor postnatal glucose levels in the infants and such mechanism cannot be further explored in the present dataset. Another possible mechanism behind the impaired cVEPs is iron deficiency. It has been well shown that infants born to diabetic mothers are at increased risk of iron deficiency,[42] which is correlated to impaired neurodevelopment. In a subsample of the present cohort, we measured iron status in cord blood and found no lower iron stores in infant born to the GD mothers.[19] Finally, it has been suggested that infants born to diabetic mothers are at high risk of hypomagnesemia.[43] Magnesium plays an important role in a wide variety of critical cellular processes including carbohydrate metabolism. Magnesium depletion, particularly in the hippocampus, has been associated to impaired cognitive development and cerebral palsy.[44] Unfortunately, maternal or infant magnesium was not assessed in the present study and we could not analyze its impact on the results.

An interesting observation was that the differences in latencies, most likely correlating to the degree of neuronal myelination, was not significant at 3 months but at 18 months. Neuronal myelination is an ongoing process during the first two years of life and the results suggests that the negative effect that follows GD has a negative impact on the myelination, also during the postnatal brain development. However, the non-significant effect at 3 months may also correlate to difficulties of assessing this outcome at such a low age.

The correlations observed between cVEPs and cognitive scores are similar to previous studies. Nelson et al. reported that cVEPs technique correlated to memory deficits in children.[35] We have previously reported no significant differences in Bayley scores in the infants born to GD mothers, but a trend of lower scores in the obese group at 18 months.[27] The cVEPs constitute a more objective outcome with regard to neuron function and myelination, however, it will require further long term follow-up trials to explore if cVEPs or Bayley scores in early life are good predictors of long term cognitive development.

Due to its observational design, this study was limited with regard to exploring causative correlations. Furthermore, it was limited by the large drop outs between delivery and 6 months of age. However, we used an objective neurophysiological test in a large number of participants and adjusted for several important sociodemographic confounders, making our observed correlations relevant for the research field. Furthermore, the study was strengthened by the fact that we could separately analyze the correlation to gestational diabetes and overweight, and obesity respectively. Nevertheless, the observation about poor cVEPs in GD mothers’ offspring requires confirmative and larger studies. Furthermore, it is relevant to further explore the interaction with maternal overweight and obesity.

In conclusion, infants born to mothers with GD had less developed cVEPs at 18 months, suggesting a suboptimal neurodevelopment. We hypothesize that the mechanism behind this observation is a poor maternal metabolic control causing damage to the developing brain in the fetus. Furthermore, our results suggest a negative interaction with maternal obesity/overweight indicating that the double burden of high pre-gestational BMI and GD causes increased risk. Moreover, cVEPs measures correlated to the Bayley scores at 18 months of age, supporting the hypothesis that cVEPs are promising a proxy for cognitive development in infancy.

## Supporting information

S1 Table cVEP results at 3 months and 18 months. (XLSX)
